# Effectiveness, safety and acceptability of ‘see and treat’ with cryotherapy by nurses in a cervical screening study in India

**DOI:** 10.1038/sj.bjc.6603633

**Published:** 2007-02-20

**Authors:** R Sankaranarayanan, R Rajkumar, P O Esmy, J M Fayette, S Shanthakumary, L Frappart, S Thara, J Cherian

**Affiliations:** 1 Screening Group, International Agency for Research on Cancer, 150 cours Albert Thomas, Lyon 69008, France; 2 Preventive Medicine Department, PSG Medical College, Coimbatore, 641004 Tamil Nadu, India; 3 Department of Radiotherapy, Christian Fellowship Community Health Centre, Ambillikai, Dindigul District, Tamil Nadu 624612, India; 4 Department of Pathology, PSG Institute of Medical Sciences and Research, Coimbatore 641 004, Tamil Nadu, India; 5 Laboratoire d'Anatomie et de Cytologie Pathologiques, Hôpital Edouard Herriot, Lyon 69003, France; 6 Department of Pathology, Regional Cancer Centre, Medical College Campus, Trivandrum, Kerala 695011, India

**Keywords:** cervical cancer, screening, early detection, cervical intraepithelial neoplasia, ‘see and treat’, cryotherapy

## Abstract

We evaluated a ‘see and treat’ procedure involving screening, colposcopy, biopsy and cryotherapy by trained nurses in one-visit in field clinics in a cervical screening study in South India for its acceptability, safety and effectiveness in curing cervical intraepithelial neoplasia (CIN). Women positive on visual inspection with acetic acid (VIA) were advised colposcopy, directed biopsies and cryotherapy if they had colposcopic impression of CIN in one visit by nurses in field clinics supervised by a doctor. Side effects and complications were assessed and cure rates were evaluated with VIA, colposcopy and biopsy if colposcopic abnormalities were suspected. Cure was defined as no clinical or histological evidence of CIN at ⩾6 months from treatment. Of the 2513 women offered ‘see and treat’ procedure, 1879 (74.8%) accepted. Of the 1397 women with histologically proved CIN treated with cryotherapy, 1026 reported for follow-up evaluation. Cure rates were 81.4% (752 out of 924) for women with CIN 1; 71.4% (55 out of 77) for CIN 2 and 68.0% (17 out of 25) for CIN 3. Minor side effects and complications were documented in less than 3% of women. ‘See and treat’ with cryotherapy by nurses under medical supervision is acceptable, safe and effective for cervical cancer prevention in low-resource settings.

Cryotherapy offers an effective and simple outpatient treatment for women with cervical intraepithelial neoplasia (CIN) (Alliance for Cervical Cancer Prevention, 2003; Sellors and Sankaranarayanan, 2003). The currently available data on its effectiveness, safety and acceptability are based on treatment provided by medical doctors in developed countries (Alliance for Cervical Cancer Prevention, 2003). The evidence from such published studies indicate that cryotherapy is an effective, safe and acceptable treatment and cure rates exceed 86% for women with CIN confined to the ectocervix (Alliance for Cervical Cancer Prevention, 2003). However, it is not clear if similar cure rates can be achieved for cryotherapy provided by trained nurses in field conditions in less developed countries.

‘See and treat’ usually involves a loop electrosurgical excision procedure (LEEP) simultaneously to diagnose and to treat pre-malignant cervical disease in screen-positive women in one visit thereby eliminating the need of a second visit for treatment (Ferris *et al*, 1996). We adapted the ‘see and treat’ principles to achieve maximum compliance of screen-positive women for diagnosis and cryotherapy in one visit within the context of a randomised controlled screening trial in South India designed to evaluate the impact of visual inspection with acetic acid (VIA) on cervical cancer incidence and mortality (Sankaranarayanan *et al*, 2004). The screening trial, a collaborative research project of the Christian Fellowship Community Health Centre (CFCHC), Ambillikai, in Dindigul district, Tamil Nadu, India and the International Agency for Research on Cancer (IARC), Lyon, France, was reviewed and approved by the institutional ethical review committees of both CFCHC and IARC. The design, methodology and the preliminary results of the trial have been published elsewhere (Sankaranarayanan *et al*, 2004, 2005). In this paper, we describe the acceptability, safety and effectiveness of ‘see and treat’ procedure involving screening, colposcopy, directed biopsy and double-freeze cryotherapy by trained nurses in a single-visit in field clinics supervised by a doctor.

## MATERIALS AND METHODS

### Participants

Apparently healthy women aged 30–59 years, with an intact uterus and no past history of cervical neoplasia in clusters of villages randomised to the screening group were identified by active enumeration of households. Female health workers explained the study and the risk factors, prevention, early detection and treatment of cervical cancer to eligible women. A printed consent form was read out and signature, or left thumb impression, of willing eligible women was obtained in the presence of a witness.

### Training of nurses in VIA, colposcopy and cryotherapy

Eight registered nurses with 3 years' nursing education learned VIA, colposcopy and cryotherapy in a 3-week intensive training course, using manuals prepared by the IARC (Sankaranarayanan and Wesley, 2003; Sellors and Sankaranarayanan, 2003). Training included lectures, discussions, review of photographs of normal and abnormal cervix, and clinical sessions to observe and practice VIA, colposcopy, directing biopsy and cryotherapy, initially under the supervision of the teaching faculty and then independent assessment with the findings checked by the faculty.

The training allowed the nurses to develop skills in obtaining gynaecological history, visualising the cervix to recognise the squamocolumnar junction (SCJ), transformation zone (TZ), assessing the TZ with acetic acid and Lugol's iodine, in recognising acetowhite and mustard-yellow lesions, directing biopsies, in discussing results and management plans with women, in performing cryotherapy and in counseling women on follow-up care. Their performance was routinely monitored by regular review of screen-positive rates, correlation between colposcopy and histology findings, and routine medical supervision in the field. Refresher courses were conducted every 3 months to maintain competency.

### Screening, colposcopy and directed biopsy

The women were invited for VIA screening in field clinics organised in village primary health centres, municipal offices, schools, or women's club buildings. A medical doctor (gynaecologist or clinical oncologist or general practitioner) was always present and supervised the functioning of the field clinic. The nurses consulted the doctor whenever in doubt or if they required assistance. During screening clinics the nurse exposed and examined the cervix using a speculum and bright halogen focus lamp. Acetic acid (4%) was applied to the cervix using a cotton swab and VIA findings were reported 1 min after the application as negative or positive (Sankaranarayanan and Wesley, 2003). Test results were explained to women and VIA-negative women were reassured and medication was prescribed if they suffered from cervical inflammation.

Nurses offered VIA-positive women immediate ‘see and treat’ involving colposcopy, directed biopsy for women with colposcopic impression of precancerous lesions followed by treatment with cryotherapy. Colposcopy was carried out with a binocular colposcope using 6–12 magnification, under the supervision of a medical officer. The nurses followed the steps described in the IARC manual on colposcopy (Sellors and Sankaranarayanan, 2003). If the SCJ was not visible in its entirety, even after manipulation of the endocervical canal, colposcopy was termed as unsatisfactory and those women were referred to doctors for further assessment.

In women with visible SCJ, a colposcopic impression was made in terms of normal, benign abnormalities (such as ectropion, cervicitis, atrophy, polyp), low- or high-grade lesion and invasive cancer, based on colour tone, margins and surface characteristics of acetowhite lesions, vascular features, such as punctuation, mosaics in the acetowhite lesions; atypical vessels, growth if any and appearance following application of Lugol's iodine (Sellors and Sankaranarayanan, 2003). Punch biopsies were obtained from the worst of any abnormal areas under colposcopic guidance.

### Cryotherapy

Women with colposcopic impression of low- or high-grade lesions were advised immediate cryotherapy after a directed biopsy, when all the following criteria were met:

- The lesion involved less than three quadrants of the TZ;
- No extension of the lesion into the endocervical canal;
- No extension of the lesion onto the vaginal walls;
- Entire lesion could be covered by the cryoprobe
- SCJ was visible in its entirety;
- No clinical or colposcopic suspicion of invasive cancer.

Women with extensive ectropion or chronic cervicitis without colposcopic features of CIN were also encouraged to undergo cryotherapy, after taking a punch biopsy. Women with lesions not eligible for cryotherapy were referred to the CFCHC for LEEP or cold knife conisation.

Before treatment, nurses explained colposcopy results and cryotherapy procedure, potential benefits, side effects and complications and encouraged the woman to accept immediate treatment. Cryotherapy was performed using nitrous oxide refrigerant, with a 20–24 mm ectocervical cryoprobe tip with a shallow nipple by standard double-freeze technique as described in the IARC manual (Sellors and Sankaranarayanan, 2003). Cryotherapy equipment, made in India, was used. No local anaesthesia, sedation or analgesics were used.

The cryoprobe tip, washed with saline, was firmly applied on the cervix to ensure adequate thermal contact. Cryotherapy involved two sequential freeze–thaw cycles, each one consisting of a 3-min freeze followed by 5 min of thawing (3-min freeze, 5-min thaw, 3-min freeze). Ice ball formation on the cryoprobe and on the cervix during the procedure was closely observed to ensure the tip did not inadvertently contact and freeze any part of the vagina during the procedure.

Once the second freeze for 3 min was completed, adequate time was allowed for thawing before removing the probe from the cervix by gentle semicircular movements. The cervix and vagina were then examined for any bleeding, particularly from the biopsy site or for any accidental vaginal freezing. The vagina was not packed with gauze after cryotherapy to allow secretions to escape. Women were provided with a supply of sanitary pads to prevent staining their clothes.

### Supportive care and instructions following cryotherapy

Women were given home-care instructions and were informed that they could experience some mild cramps and a clear or lightly bloodstained watery discharge for up to 4–6 weeks after treatment. They were advised not to use vaginal douche or tampons, or to have sexual intercourse for 1 month after treatment. They were advised to report back to the clinic if they experienced any of the following symptoms during the 4 weeks after treatment: fever ⩾2 days, severe lower abdominal pain, foul-smelling greenish yellow discharge, bleeding with clots or bleeding for over 2 days. All treated women received presumptive antibiotic treatment with oral metronidazole and doxycycline for 5 days. They were prescribed oral paracetamol for any mild pain or cramps experienced after treatment.

### Processing of biopsy and histology reporting

Biopsy specimens were processed in the screening project pathology laboratory and the slides were reported on by the pathologists at the PSG Institute of Medical Sciences and Research, Coimbatore, India. As part of quality assurance, processing of specimens in the laboratory, laboratory manuals, and reporting procedures were periodically reviewed, and technicians and pathologists were re-trained. A sample of slides was reviewed for reproducibility by pathologists from the Regional Cancer Centre, Trivandrum, India and University of Lyon, France.

### Follow-up of women treated with cryotherapy

Each woman treated for CIN was given an appointment for a follow-up visit 1-year after treatment to assess the cervix and to rule out cervical neoplasia, but some reported early for follow-up evaluation. All those who reported earlier than 6 months after treatment and never came afterwards were considered as ‘lost to follow-up’. At follow-up clinics, again supervised by medical doctors, VIA and colposcopy were carried out by nurses and biopsies were directed from colposcopically abnormal areas. Women with confirmed disease at follow-up were treated with repeat cryotherapy or excision treatment depending upon the lesion characteristics.

### Evaluation of outcomes: cure rates, side effects and complications

The outcome of evaluation at follow-up was defined as ‘cure’ if no CIN lesions were established histologically if a biopsy specimen was obtained or no colposcopic features of CIN were demonstrated in cases without biopsy.

Mild pain or mild cramps during or after treatment, fainting and flushing during or immediately after treatment, mild bleeding or spotting immediately after treatment or malodorous excessive discharge following treatment were assumed to be side effects and as an indicator of acceptability (Alliance for Cervical Cancer Prevention, 2003). Anaphylactic reactions during treatment, severe pain, cramps or severe bleeding during or after cryotherapy requiring further treatment, severe local cervical infections, unintended surgery within 4 weeks following cryotherapy, pelvic inflammatory disease, and functional cervical stenosis were assumed to be complications of treatment and as an index of safety of treatment (Alliance for Cervical Cancer Prevention, 2003). Cure rates, side effects and complications were reported as frequency percentages. Cure rates categorised by age, area of lesion and grade of CIN were compared using *χ*^2^ statistic.

### Role of the funding source

The funding source had no role in study design, data collection, analysis and interpretation, writing of the report, or in the decision to submit the report for publication.

## RESULTS

Of the 2513 women treated with cryotherapy in our study, 1879 (74.8%) had it on the same day as screening, colposcopy and directed biopsy; the remaining wanted to consult their family: 233 (9.3%) returned within 2 weeks to receive treatment, whereas 401 (15.9%) availed treatment after 2 weeks from screening and colposcopy.

**Figure 1:**
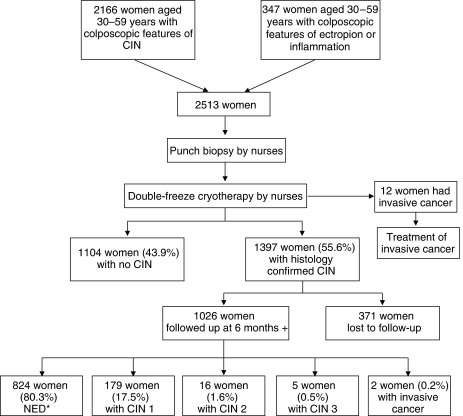
Flow chart of study procedures and results. ^*^NED: no evidence of disease (cured).

Colposcopic impression and histology of the 2513 women treated with cryotherapy are given in Table 1 (Figure 1): 1397 (55.6%) had CIN (1242 CIN 1 and 155 CIN 2–3) on histology. The 12 women with subclinical invasive cancer who received cryotherapy were later referred for cancer treatment. Of the entire 1573 women with histologically confirmed CIN, 1397 (88.8%) had cryotherapy, 88 (5.6%) had LEEP and only 82 (5.2%) did not avail treatment in our study.

The distribution of follow-up times from the date of cryotherapy for evaluation of cure rates are given in Table 2: 1026 women (73%) reported for follow-up between 6 and 56 months and were included for analysing the cure rates, side effects and complications of cryotherapy; 371 (27%) were lost to follow-up (Figure 1). The mean follow-up duration was 27 months. The disease status at follow-up was based on histology in 595 (58.0%) women and on colposcopy in the remaining 431 (42.0%) women. The distribution of follow-up status is given in Table 3: 824 (80.3%) women had no evidence of CIN and were considered cured; the cure rate for women with CIN 1 was 81.4 and 70.6% for women with CIN 2–3 lesions (p 0.009).

The follow-up status among 885 (86.3%) VIA-negative women at follow-up evaluation is as follows: 789 were cured; CIN 1, 87; CIN 2–3, 9; among 141 (13.7%) VIA-positive women: 35 were cured; CIN 1, 92, CIN 2–3, 12; invasive cancer: 2. The sensitivity, specificity, positive and negative predictive values of VIA detecting CIN 2 or worse lesions at follow-up were 60.9, 87.3, 9.9 and 99.0%, respectively. Cure rates of CIN according to age, area of cervix involved and grade of the lesion are given in Table 4. Women with CIN 2–3 had a significantly lower cure rate compared to those with CIN 1. Age and area of lesion did not affect cure.

The following side effects were observed: mild pain or mild cramps during or after treatment in 25 (2.1%) women; malodorous excessive vaginal discharge following treatment in 19 (1.6%); mild bleeding or spotting in nine (0.7%), delayed bleeding from the biopsy site in three (0.2%), and fever in two (0.2%) women. The following complications were recorded: local cervical infection in 14 women (1.4%), cervical tenderness in five (0.5%), severe pelvic cramps and lower abdominal pain requiring parenteral medication in one (0.1%) and one (0.1%) suffered from accidental vaginal freezing. There were no instances of anaphylactic reactions, severe bleeding requiring suturing or blood transfusion, unintended surgeries or pelvic inflammatory disease requiring hospitalisation or functional cervical stenosis.

## DISCUSSION

Our findings have important implications for efficient service delivery and cancer prevention in cervical screening programmes in low- and medium-resourced settings. It indicates that nurses, after adequate in-service training and under medical supervision, can effectively perform screening, colposcopy, directing biopsies, providing cryotherapy and follow-up evaluation.

Our findings confirm a ‘see and treat’ approach using cryotherapy by nurses is acceptable to women, safe and ensures satisfactory participation of screen-positive women for diagnosis and treatment. The ‘see and treat’ approach has been mainly responsible for the high proportion of women with CIN receiving treatment. The acceptance by three out of four women offered the single visit ‘see and treat’ procedure and the fact that only a negligible proportion of women with histologically confirmed CIN did not avail treatment amply demonstrate the high acceptability and usefulness of this approach in maximising treatment coverage. It has been our policy to treat women with low-grade disease, as our intervention is a once in a life-time and due to difficulties in ensuring follow-up. Directed biopsy, taken just before cryotherapy, has proved to be safe and has given a unique opportunity to overcome two important limitations of cryotherapy, namely, lack of histological proof of lesions treated and the risk of ignoring those with occult invasive cervical cancers who received suboptimal treatment with cryotherapy.

That 60% of women with a colposcopic impression of a precancerous lesion had a histologically confirmed CIN indicates that nurses can provide reasonably competent colposcopy under medical supervision. ‘See and treat’ with cryotherapy in our study resulted in 40% of the women being unnecessarily treated and 12 (0.6%) had an occult invasive cancer frozen. The availability of histology after treatment enabled these women to be treated adequately. Even though, one fourth of the treated women could not be evaluated for cure due to loss to follow-up, given the difficulties in tracing women in developing countries, our follow-up rates are satisfactory.

Adequate treatment of CIN is vital for the success of cervical screening in preventing cervical cancer. Existing cervical screening programmes in low- and medium-resource countries have been less successful in reducing cervical cancer burden partly owing to inadequate coverage of treatment of women detected with CIN (Sankaranarayanan *et al*, 2001; IARC, 2004). Lack or inadequacy of well-trained medical manpower for colposcopy and treatment of CIN is a major resource constraint in many developing countries (Sankaranarayanan *et al*, 2001). Well-trained nurses provide alternative human resources for screening, colposcopy and treatment by cryotherapy (Gifford and Stone, 1993; Hartz, 1995; Todd *et al*, 2002; Royal Thai College of Obstetricians and Gynaecologists (RTCOG)/JHPIEGO Corporation Cervical Cancer Prevention Group *et al*, 2003; Sankaranarayanan *et al*, 2004; McPherson *et al*, 2005; Gage *et al*, 2006). There is some evidence that colposcopy provided by nurses is as good as doctors and that nurses are viable alternate providers (Gifford and Stone, 1993; Hartz, 1995; Todd *et al*, 2002; McPherson *et al*, 2005; Gage *et al*, 2006). Nurse colposcopists have been introduced in countries such as the US, UK and New Zealand in recent years.(Gifford and Stone, 1993; Hartz, 1995; Todd *et al*, 2002; McPherson *et al*, 2005; Gage *et al*, 2006).

There is enough evidence that cryotherapy provided by doctors is an effective and safe procedure for treating CIN (Alliance for Cervical Cancer Prevention, 2003). However, there is only limited information on the effectiveness of cryotherapy, provided by trained nurses in field conditions in developing countries. In Thailand, nurses provided cryotherapy for VIA-positive women in a study evaluating ‘single visit approach’ (SVA), but the histological nature of the treated lesions was not available as colposcopy and biopsy were not provided in this study (Royal Thai College of Obstetricians and Gynaecologists (RTCOG)/JHPIEGO Corporation Cervical Cancer Prevention Group *et al*, 2003). However, combining VIA and cryotherapy in a single sitting was found to be feasible, safe and acceptable to women (Royal Thai College of Obstetricians and Gynaecologists (RTCOG)/JHPIEGO Corporation Cervical Cancer Prevention Group *et al*, 2003).

Our experience indicates that four-fifths of women treated using cryotherapy by nurses were cured and cure rates for those with high-grade CIN lesions were significantly lower than those for low-grade lesions. Our overall cure rate was slightly lower than the summary 89.5% overall cure rate found in a recent review of 32 studies (seven randomised controlled trials and 25 case studies) predominantly conducted in developed countries and in which cryotherapy was provided by experienced doctors; higher cure rates were found for CIN 1 lesions, smaller lesions and among those included in randomised trials; less than 20% of the women were lost to follow-up in studies included in this review (Alliance for Cervical Cancer Prevention, 2003). The somewhat lower cure rates for cryotherapy by nurses in our study may be due to a number of reasons. More stringent assessment of lesions at follow-up as evidenced by the fact three out of five evaluated women had a biopsy possibly resulted in a large proportion of failures correctly established. Essentially prevalent lesions among our previously unscreened women were treated. There might be a learning curve in effectively providing the treatment.

We observed side effects and complications following cryotherapy in only a negligible proportion of women in our study and none were major or life threatening, which is similar to those reported in other studies (Alliance for Cervical Cancer Prevention, 2003). Our results indicate that ‘see and treat’ cryotherapy provided by nurses in field conditions is safe and acceptable to women and support the fact that one can safely direct biopsy before giving cryotherapy as evidenced by the very low frequency of delayed bleeding from the biopsy site. Biopsies directed before cryotherapy provide a safeguard against freezing and leaving occult invasive cancers without further intervention.

In summary, from a public health perspective, our results have important implications for cervical cancer screening programmes in developing countries. Our results provide important evidence that adequately trained nurses can be used to deliver colposcopy and cryotherapy services in cervical screening programmes and are important, reliable, and efficient alternate human resources. However, the quality of their inputs and outcome should closely and continuously be monitored to ensure good quality services.

## Figures and Tables

**Table 1:** Colposcopy and histology findings of 2513 women treated with cryotherapy

	**Histology at baseline (before cryotherapy)**					
**Colposcopy impression at enrolment**	**No evidence of CIN^a^**	**CIN 1**	**CIN 2**	**CIN 3**	**Cancer**	**Total**
Ectropion or cervicitis	260	81	3	3	0	347
Low-grade lesion	786	1051	87	31	8	1963
High-grade lesion	58	110	22	9	4	203
Total	1104	1242	112	43	12	2513

a Cervical intraepithelial neoplasia.

**Table 2:** Details of follow-up of women with CIN treated with cryotherapy

	**Histology at baseline (before cryotherapy)**			
**Follow-up after cryotherapy**	**CIN^a^ 1**	**CIN 2**	**CIN 3**	**Total**
No follow-up	316	35	18	369
Less than 6 months	2	0	0	2
7 Months or more	924	77	25	1026
Total	1242	112	43	1397

a Cervical intraepithelial neoplasia.

**Table 3:** Follow-up status based on histology or colposcopy (range 7–57 months, mean follow-up 27 months)

		**Follow-up status**				
**Histology at baseline before cryotherapy**	**Number of women evaluated**	**No evidence of disease (cured %)**	**CIN^a^ 1 (%)**	**CIN 2 (%)**	**CIN 3 (%)**	**Invasive cancer (%)**
CIN 1	924	752 (81.4)	162 (17.5)	7 (0.8)	1 (0.1)	2 (0.2)
CIN 2	77	55(71.4)	14 (18.2)	7 (9.1)	1 (1.3)	0
CIN 3	25	17 (68.0)	3 (12.0)	2 (8.0)	3 (12.0)	0
Total	1026	824 (80.3)	179 (17.5)	16 (1.6)	5 (0.5)	2 (0.2)

a Cervical intraepithelial neoplasia.

**Table 4:** Cure rates at follow-up according to characteristics of women at screening

**Characteristics**	**Cure rate**	***P*-value for *χ*^2^ independence test**
*Age*		0.29
30–39	80.9% (683/844)	
40–55	77.5% (141/182)	
		
*Area of involvement of cervix by the lesion* a		0.12
<25%	81.2% (696/857)	
25%	75.8% (116/153)	
		
*Grade of CIN* b		0.009
CIN 1	81.4% (752/924)	
CIN 2–3	70.6% (72/102)	

a Missing in 16 cases.

b Cervical intraepithelial neoplasia.
